# Understanding Cognitive Complaints Among Breast Cancer Survivors

**DOI:** 10.1093/geroni/igaa057.1916

**Published:** 2020-12-16

**Authors:** Brent Small, Heather Jim, Sarah Eisel, Stacey Scott

**Affiliations:** 1 University of South Florida, Tampa, Florida, United States; 2 Moffitt Cancer Center, Tampa, Florida, United States; 3 Stony Brook University, Stony Brook, New York, United States

## Abstract

Cancer and its treatment can induce accelerated aging changes in physiological and behavioral processes. In studies of cancer associated cognitive decline, subjective reports of cognitive impairment are often many times greater than performance deficits on objective tests of neurocognitive functioning. In an Ecological Momentary Assessment study of 47 breast cancer patients (M age = 53.3 years), subjective ratings of cognitive performance and the occurrence of memory lapses assessed at the end of day were predicted by cognitive performance and ratings of fatigue and depressed mood throughout the day. Results indicated that poorer subjective cognition was significantly associated with elevated fatigue throughout the day. Slower processing speed, elevated ratings of fatigue, and depressed mood throughout the day were associated with a greater likelihood of memory lapses. Subjective ratings of cognitive deficits are related to objective performance, as well as common quality of life decrements among cancer survivors.

